# Linkage of multiple electronic health record datasets using a "spine linkage" approach compared to all "pairwise linkages".

**DOI:** 10.23889/ijpds.v7i3.2070

**Published:** 2022-08-25

**Authors:** Helen Blake, Linda Sharples, Katie Harron, Jan van der Meulen, Kate Walker

**Affiliations:** 1 London School of Hygiene and Tropical Medicine; 2 Royal College of Surgeons of England; 3 University College London (UCL) Great Ormond Street Institute of Child Health

## Objectives

To compare two approaches for linking multiple datasets: using all "pairwise linkages", linking each dataset to every other dataset; versus linking each dataset to a designated "spine dataset", by: considering the differences between these approaches, and illustrating using real-world data on patients undergoing emergency bowel cancer surgery.

## Approach

We linked an administrative hospital dataset (Hospital Episode Statistics; HES) capturing patients admitted to hospitals in England, and two clinical datasets comprising patients undergoing emergency bowel surgery (National Emergency Laparotomy Audit; NELA) and patients diagnosed with bowel cancer (National Bowel Cancer Audit; NBOCA), with study period from 31 October 2013 to 30 April 2018. We compared pairwise linkage to spine linkage, designating HES as the spine dataset, by considering the number of eligible patients linked by each approach, characteristics of linked patients, levels of missing data, and whether analysis results were sensitive to the approach used.

## Results

The spine linkage approach resulted in an analysis cohort of 15,826 patients, equating to 98.3% of the 16,100 patients identified with the pairwise linkage approach. Of 274 additional patients captured in the pairwise approach, approximately two-thirds were only in the emergency surgery dataset (NELA) and one-third were only in the bowel cancer dataset (NBOCA). There were no systematic differences in patient characteristics between these analysis cohorts. Associations of patient and tumour characteristics with mortality, complications, and length of stay were not sensitive to the linkage approach. When eligibility criteria were applied before linkage, spine linkage included 14,509 patients (90.0% compared to pairwise linkage).

## Conclusion/Implications

Spine linkage can be an efficient alternative to pairwise linkage, if case ascertainment in the spine dataset and data quality of linkage variables are high. These aspects should be systematically evaluated in the nominated spine dataset before spine linkage. Results are sensitive to order of linkage steps.

